# Effects of patient load and travel distance on HIV transmission in rural China: Implications for treatment as prevention

**DOI:** 10.1371/journal.pone.0177976

**Published:** 2017-05-31

**Authors:** M. Kumi Smith, William C. Miller, Huixin Liu, Chuanyi Ning, Wensheng He, Myron S. Cohen, Ning Wang

**Affiliations:** 1 Department of Epidemiology, University of North Carolina Chapel Hill, Chapel Hill, North Carolina, United States of America; 2 Ohio State University College of Public Health, Columbus, Ohio, United States of America; 3 Department of Clinical Epidemiology, Peking University People's Hospital, Beijing, People’s Republic of China; 4 University of North Carolina Institute for Global Health and Infectious Diseases, University of North Carolina, Chapel Hill, North Carolina, United States of America; 5 Zhumadian City Centers for Disease Control, Zhumadian, Henan, People’s Republic of China; 6 National Centre for AIDS/STD Control and Prevention, China Centers for Disease Control and Prevention, Beijing, People’s Republic of China; University of Queensland, AUSTRALIA

## Abstract

**Background:**

Sustained viral suppression through ART reduces sexual HIV transmission risk, but may require routine access to reliable and effective medical care which may be difficult to obtain in resource constrained areas. We investigated the roles of patient load and travel distance to HIV care clinic on transmission risk in HIV serodiscordant couples in Henan Province, China.

**Methods:**

Cox proportional hazard models were used to compare HIV transmission events across couples living near, medium, or farther distances from their assigned HIV care clinics, as well as those attending clinics where clinicians bore high versus low patient loads.

**Results:**

Most (84·4%) of the 3695 serodiscordant couples lived within 10 kilometers of their assigned HIV clinic, and most (73·5%) attended clinics with patient-to-provider ratios of at least 100:1. In adjusted Cox models, attending clinics where clinicians bore average patient loads of 100 or more elevated HIV transmission risk (aHR, 1·50, 95% CI, 1·00–4·84), an effect amplified in village tier clinics (aHR = 1·55; 95% CI, 1·23–6·78). Travel distance was associated with HIV transmission only after stratification; traveling medium distances to village clinics (5-10km) increased transmission risk (aHR = 1·83, 95% CI, 1·04–3·21) whereas traveling longer distances to township or county level clinics lowered transmission risk (aHR = 0·10, 95% CI, 0·01–0·75).

**Conclusion:**

Higher patient loads at HIV clinics was associated with risk of HIV transmission in our population, particularly at village level clinics. Farther travel distance had divergent effects based on clinic tier, suggesting unique mechanisms operating across levels of resource availability. The resource intensity of long-term HIV treatment may place significant strains on small rural clinics, for which investments in additional support staff or time-saving tools such as point-of-care laboratory testing may bring about impactful change in treatment outcomes.

## Introduction

Universal antiretroviral therapy (ART) coverage is a central pillar of the global HIV agenda,[1–3] fueled by the twin goals of equitable drug access and HIV disease eradication through transmission prevention.[4] ART coverage rates continue to improve on a global scale;[5] yet access remains uneven resulting in higher transmission rates and poorer treatment outcomes in lower income and more socially marginalized HIV patients. The success of a global treatment as prevention strategy will depend on its ability to adapt to diverse geographies, healthcare environments, and resource availability.

“Implementation science” is a multidisciplinary field that seeks to close the gap between interventions proven to work in trial settings and effective delivery of these tools in the real world.[6] Past research on HIV treatment outcomes in community settings have found that patient access to routine healthcare is a key determinant sustained viral suppression, suggesting these factors may also shape the efficacy of ART as a means of prevention. Reports from the US and sub-Saharan Africa, for example, find that weaker patient-provider relationships[7–9] or poorer physical access to care clinics[10–13] predict greater loss of HIV patient follow-up and poorer treatment adherence in treated patients. Similarly in China, tier of HIV care (village clinics versus township or county level hospitals) has predicted higher rates of virological failure[14] and drug resistance.[15,16]

Such findings underscore the importance of the treatment environment in determining HIV transmission patterns in settings where ART coverage is already quite high.[17] To explore the relationship between treatment access and transmission in such settings, we used available data from a well-studied cohort of HIV serodiscordant couples[18–20] in China, where ART is free and universal.[21] By measuring the impact of factors such as patient load and travel distance on the risk of sexual HIV transmission between partners, this analysis provides a better understanding of the role of the healthcare environment in the preventive utility of ART.

## Methods

### Study setting and population

The study cohort arises from a population of HIV infected individuals in central China where regional blood selling scandals in the 1990’s led to mass HIV transmission of up to 30,000 people, mostly poor farmers, through unsanitary blood collection practices.[22] In response, the national government established a free national ART program in 2002, in which HIV infected individuals are eligible to receive, free of charge, the following: first-line ART, annual viral load and quarterly CD4 testing, and coverage for a predetermined list of essential medicines for common opportunistic infections associated with HIV [17]. The program has been noted for its rapid roll out and success—mortality in treated HIV patients fell from 39·3 to 14·2 deaths per 100 person-years between 2000 and 2009.[17] Within Henan province, the epicenter of the blood selling HIV epidemic, over one-third of HIV-infected persons live in the prefecture of Zhumadian, where the data used in this analysis were collected.[23]

Success of China’s ART program has been attributed to its exploitation of the existing three-tier healthcare system (Table 1), in which a subset of facilities in each county is designated to specialize in HIV care based on the geographical distribution of patients.[24] Patients access free ART and related medical care at their assigned HIV care clinics, which may be a village, town, or county level site depending on their county’s delivery strategy. Severe cases may be transferred up the chain of referral if more specialized care is needed; however, rates of government subsidization fall significantly for care received at each tier above the patient’s assigned care clinic. Independent care seeking outside of this referral network is possible; however, patients who do so bear the entirety of incurred medical costs themselves.

**Table 1:** Feature of the three tiers of healthcare entities providing HIV care in Zhumadian.

	Village clinic	Township health center	County level hospital-based clinics
Services	Basic health services including physical examination and drug dispensation by non-physician clinicians.	Primary healthcare and supervision of village clinics. Full time pharmacists.	Larger medical center with referral and specialty services. Full time pharmacists.
Medical Staff	One full-time or several part-time non-physician clinicians.	Several full time physicians.	Full time physician clinicians; usually staffed within an infectious disease ward.
Laboratory testing capacity	No on site laboratory; all lab samples are transported to higher level laboratories for testing.	Some CD4 cell count monitoring capacity; no VL monitoring capacity.	Full CD4 and VL monitoring capacity.

Following a mass HIV screening campaign in 2004, local disease control centers identified what were thought to be the majority of HIV infected persons in the study prefecture, and began follow-up of those with uninfected spouses in order to monitor HIV transmission events. Eligible couples must meet the following criteria:1) registered residents living in the study prefecture of Zhumadian, 2) over 16 years of age (the age of legal consent in China), 3) in a stable marriage (no separation or divorce), 4) one partner confirmed to be HIV seropositive and the other seronegative, and 5) willing to provide informed consent. HIV status of both partners is confirmed at enrollment through enzyme-linked immunosorbent assay (ELISA, Lizhu, Zhuhai, Guangdong Province; Xinzhuang, Xiamen, Fujian Province) conducted by county-level CDC’s, and positive test results are confirmed by western blot assay (Ou’ya, Hangzhou, Zhejiang Province). This analysis considered all visits made between October 2006 and September 2012.

Enrolled cohort members participate in annual surveys with trained staff from county level disease control centers. Each partner takes part in separate face-to-face interviews to provide information on demographic characteristics and HIV-related risk factors including sexual behaviors within and without the primary partnership, history of ever having a diagnosis for sexually transmitted infections, and history of having ever injected drugs, donating blood, or undergoing a blood transfusion. Initially infected (or index) partners provide updated information on ART treatment and incidents of opportunistic infection.

### Exposure, outcome and other covariates

We assessed two primary exposures of interest: average patient load at each HIV care clinic and distance in kilometers (km) from couples’ home villages to their assigned clinics. Relevant clinic-specific information was solicited from a written survey administered to county health officials in September 2013, which collected data on numbers of medical staff and HIV/AIDS patients in follow-up at each clinic. Average patient load was then calculated as the ratio of patients to clinicians—nurses, non-physician clinicians (staff who are not trained as physicians but who are capable of many of the diagnostic and clinical functions of medical doctors), and physicians—at each HIV care clinic. Each type of coding for patient load—continuous, categorical, and quadratic spline—was plotted against predicted risk of HIV transmission to inform its final coding as a three-category variable of low (<50), medium: (≥50 and <100) and high (≥100) patients per clinician.

Distance was measured from patients’ home villages to their assigned HIV care clinics by using addresses reported in the study dataset or, where missing, from the national surveillance databases. The names of the assigned HIV care clinics were obtained from the same survey administered to county officials in 2013. Three coders (authors MKS, HXL, and CYN) used web-based geomapping software (Gaode Ditu: http://ditu.amap.com/) to manually enter origin (patient’s home village) and destination (assigned clinic) addresses in order to estimate the length in km of the most likely pedestrian path traveled between the two points. Each distance was measured by two coders, between which discrepancies of greater than 10km were resolved through discussion and remeasurement by the two coders. In all other cases the shorter distance was used. Coding of the distance variable was explored using a similar approach as with patient load, which informed our final coding as near (<5km), medium (≥5km and <10km), and far (≥10km).

The primary outcome of interest was HIV transmission, assessed from HIV antibody screening of non-index partners at annual visits. Those who screened positive were contacted for confirmatory testing, post-test counseling, and evaluation for treatment eligibility. Seroconversion was calculated as the midpoint between the date of the last HIV-negative or indeterminate test, and three months before the date of the first HIV-positive test to provide an average window period for seroconversion. Couples experiencing the outcome were censored in the interval in which estimated seroconversion occurred; those who remained discordant throughout the study were censored on the date of their last HIV-negative test date.

### Statistical analyses

Analysis was restricted to couples in which the index partner was treatment naïve at their initial visit, but who initiated ART at some point over the course of follow-up. This minimized potential bias induced by under-ascertainment of higher transmission risk earlier in patients’ treatment course, due to say, incomplete viral suppression in new ART users, and from the inability to control for baseline factors such as CD4 that are themselves affected by the treatment.[25]

Hazard ratios to assess associations between healthcare access and HIV transmission risk were estimated using Cox proportional hazard models. Plots of the hazards over time stratified by distance subgroups were examined to confirm proportionality of hazards over time, and we used directed acyclic graphs to identify the minimally sufficient set of potential confounders of the association between healthcare exposures and HIV transmission.[26] Statistical analyses were conducted in SAS 9·3 (SAS Institute Inc, Cary, NC, USA) and maps were created in ArcGIS 10·3 (Environmental Systems Research Institute, Redlands, CA, USA).

All data used for this analysis were collected as part of Zhumadian CDC local disease control efforts. Participants provided written informed consent to participate in this study, with provisions for verbal consent for illiterate participants. Signed informed consent forms are maintained by study staff and kept in a secured storage place within the local Centers for Disease Control and Prevention. Ethical approval for the analysis of this data for research purposes was provided by the Institutional Review Board of the National Center for AIDS/STD Control and Prevention (NCAIDS) at the Chinese Center for Disease Control and Prevention. This analysis relied on an agreement between the Institutional Review Boards of NCAIDS and the University of North Carolina, Chapel Hill.

## Results

Overall, 3695 treated HIV patients and their spouses contributed 21,231 person-years (PY) (Table 2). Most (84·4%) of the 3695 serodiscordant couples lived within 10 kilometers of their assigned HIV clinic, and most (73·5%) attended clinics with patient-to-provider ratios of at least 100:1. Median age of index partners was 44 years (range, 18–78) more than half (56·2%) of whom were male. About half (53.6%) of index partners (56.6% of whom were male and 43.4% of whom were female) reported blood or plasma selling as their initial route of HIV infection.

**Table 2:** Characteristics of the 3965 HIV serodiscordant couples included in the final analysis.

		HIV Transmission N (%)	No HIV Transmission N (%)	Person years	Incidence per 100 PY (95% CI)
Total		84	3611	21,231	0.4 (0.32–0.49)
Sex of index partner					
	Female	29 (34.5)	1590 (44)	9327	0.31 (0.22–0.45)
	Male	55 (65.5)	2021 (56)	11904	0.46 (0.35–0.6)
Age of index partner					
	Under 45	35 (41.7)	1895 (52.5)	11278	0.31 (0.22–0.43)
	45 and older	49 (58.3)	1716 (47.5)	9953	0.49 (0.37–0.65)
Index partner HIV transmission route					
	Blood/plasma donation	67 (79.8)	1914 (53)	10990	0.61 (0.48–0.77)
	Blood transfusion	6 (7.1)	507 (14)	3070	0.2 (0.09–0.43)
	Injection drug use	1 (1.19)	61 (1.7)	397	0.25 (0.04–1.78)
	Sexual contact	3 (3.57)	1015 (28.1)	6052	0.05 (0.02–0.15)
	Missing	7 (8.3)	114 (3.2)	772	0.91 (0.43–1.9)
Index partner occupation					
	Farmer	82 (97.6)	3327 (92.1)	19588	0.42 (0.34–0.52)
	Non-farmer	2 (2.4)	282 (7.8)	1631	0.12 (0.03–0.49)
	Missing	0 (0)	2 (0.1)	12	0 (0–0)
Clinic Type					
	Village Clinic	62 (73.8)	2238 (62)	12806	0.48 (0.38–0.62)
	Township or county hospital	20 (23.8)	1311 (36.3)	8025	0.25 (0.16–0.39)
	Missing	2 (2.4)	62 (1.7)	400	0.5 (0.13–1.99)
Distance from designated clinic*					
	Near	41 (48.8)	1791 (48.5)	10388	0.39 (0.29–0.54)
	Medium	36 (42.86)	1252 (33.9)	7037	0.51 (0.37–0.71)
	High	7 (16.28)	644 (33.8)	3806	0.18 (0.09–0.39)
	Missing	0 (0)	8 (0.2)	50	—
Patient burden of designated clinic**					
	Low	31 (56.4)	1965 (73.3)	11620	0.27 (0.19–0.38)
	Medium	24 (43.6)	717 (26.7)	4311	0.56 (0.37–0.83)
	High	29 (54.7)	929 (56.4)	5300	0.55 (0.38–0.79)
Index partner baseline CD4					
	≤250 cells/μL	42 (50)	1240 (34.3)	7442	0.56 (0.42–0.76)
	>250 cells/μL	42 (50)	2371 (65.7)	13789	0.3 (0.23–0.41)
Index partner viral suppression in first year of ART					
	Yes	34 (51.5)	1888 (78.9)	11333	0.3 (0.21–0.42)
	No	32 (48.5)	506 (21.1)	2980	1.07 (0.76–1.52)
	Missing	18 (21.4)	1217 (33.7)	6918	0.26 (0.16–0.41)

* Near: <5km; Medium: ≥5km and ≤10km; Far: >10km

** Low: <50 patients; Medium: ≥50 and ≤100 patients; Far: >100 patients

Overall, 84 HIV transmission events occurred during the study (incidence rate 0·40 cases per 100 person years; 95% confidence intervals [CI], 0·32–0·49). In unadjusted bivariable analyses, HIV transmission rates were higher for couples whose index partners had the following characteristics: male sex, infected with HIV through blood/plasma selling, had a baseline CD4 cell count below 250 cells/mm^3^, and were assigned to an HIV care clinic with more than 50 patients per clinician.

**Fig 1:**
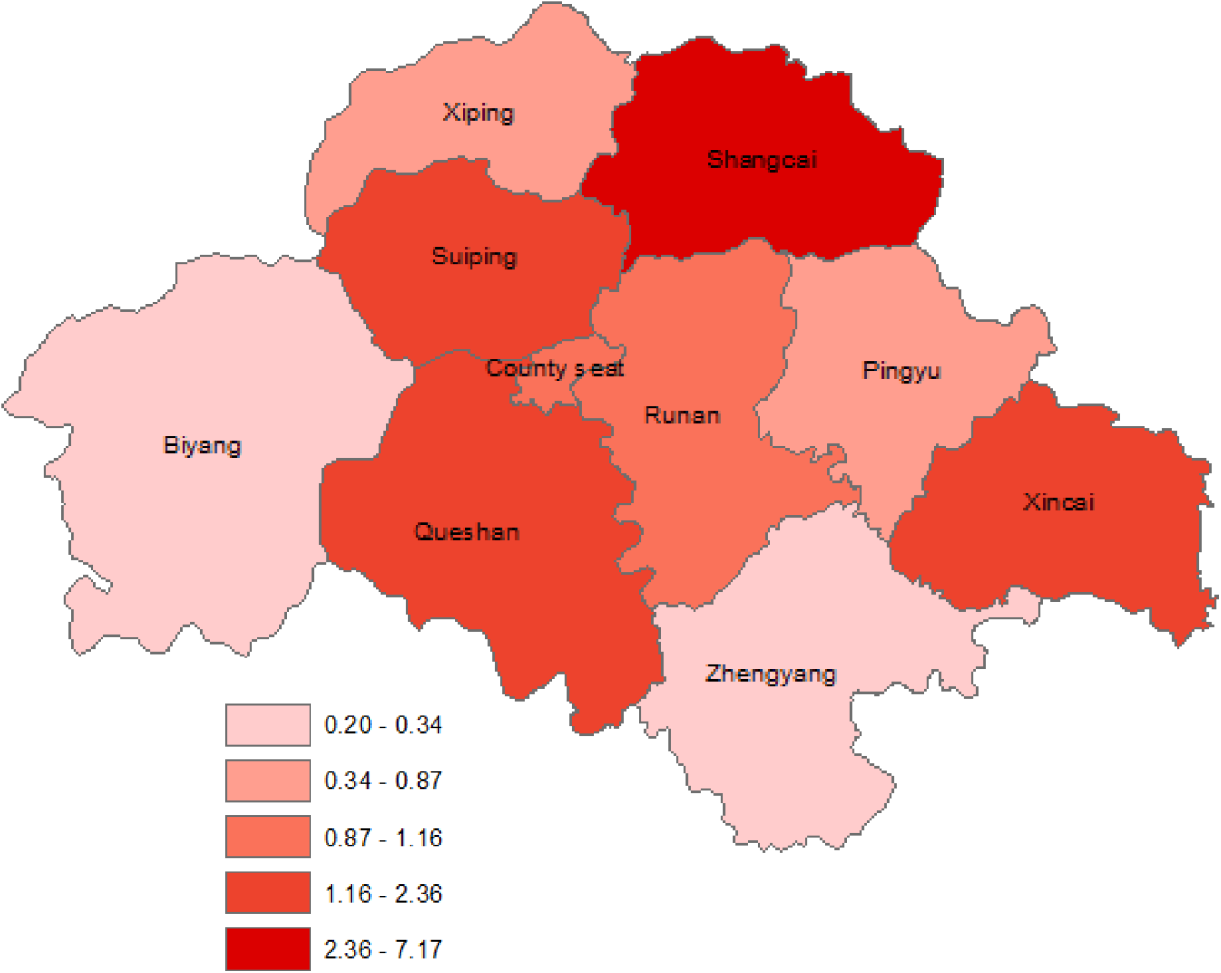
Map of the 10 counties of Zhumadian, a prefectural level city, showing county-level HIV prevalence (units: Cases per 10,000) averaged over the entire study period.

**Fig 2:**
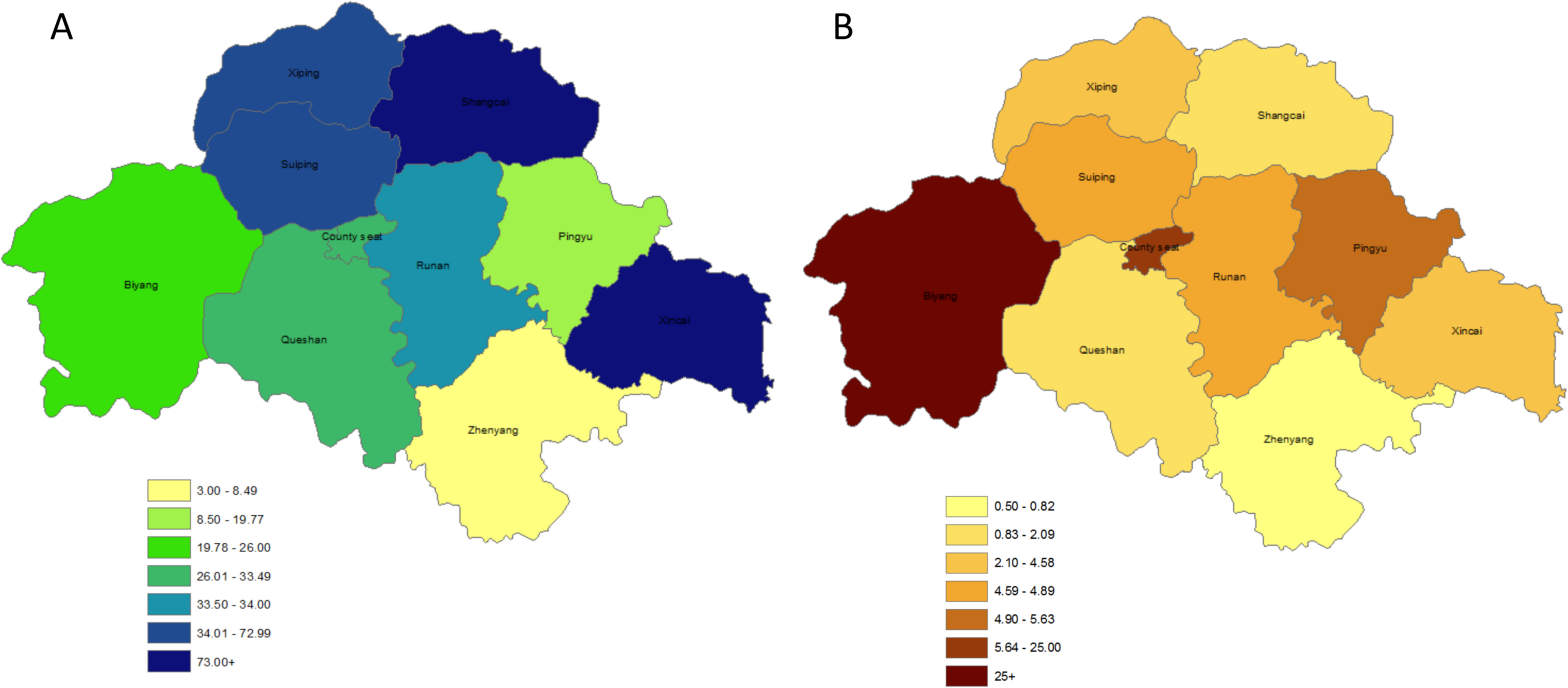
Maps of Zhumadian City showing A) median patient-to-provider ratios and B) median distance from patient homes to clinic.

Clinic tier and distribution varied widely by county (Fig 1). Most HIV care clinics in our study settings were township level healthcare clinics (N = 90), as opposed to village clinics (N = 65) or clinics based out of county hospitals (N = 4; Table 3). Median patient load per clinician varied greatly by tier of care, with the greatest load borne by clinicians at village clinics (median 48·1 patients per clinician), with similarly lower loads borne by clinicians at township and county level clinics (median 28·6 and 23·9 patients per clinician, respectively). The patient volume of hospital-based clinics was similar to village clinics, but larger staff at these upper tier centers diluted the patient load (hospital-based clinics had median staff counts of 4·5 versus 1.4 at village clinics). Patients in our cohort also lived a wide range of distances from their assigned clinics, with most (84·4%) living within 10km and a small minority (17·6%) living 10km or more away. Average distances traveled nearly doubled for each increase in tier of care (from 6·3 to 12·0 to 24·1 km), though distances varied the most among the subset traveling to county level hospital-based clinics (Fig 2).

**Table 3:** Characteristics of village, township, and county-level HIV clinics in Zhumadian City.

	Village clinic	Township health center	County level hospital-based clinic
	(N = 65)	(N = 90)	(N = 4)
	Mean (median)		
Number of treated patients per clinic	87·7 (50·0)	46·4 (34·5)	88·5 (73·0)
Number of medical staff per clinic	1·4 (1·0)	1·7 (2·0)	4·5 (3·0)
Number of patients per clinician	48·1 (32·0)	28·6 (24·0)	23·9 (23·6)
Distance traveled to clinic	6·3 (5·6)	12·0 (3·5)	24·1 (25·5)

Mean times to transmission (Table 4) varied across healthcare access exposures, and were shorter among couples assigned to HIV care at non-village clinics (2·9 versus 3·9 years), those living a medium distance from their care clinics (2·6 versus 3·5 or 4·7 years), and those assigned to clinics where clinicians faced average patient loads greater than 100 (2·8 versus 3·1 or 3·7).

**Table 4:** Mean time to transmission among couples across sub-categories of the exposures.

Subgroup		Mean time to HIV transmission in years (SD)
Tier of clinic		
	Village	3·9 (1·9)
	Town or county	2·9 (1·9)
Distance from clinic*		
	Near	3·5 (2·0)
	Medium	2·6 (1·7)
	Far	4·7 (1·8)
Patient volume**		
	Low	3·7 (2·0)
	Medium	3·1 (1·9)
	High	2·8 (1·9)

SD: standard deviation

* Near: <5km; Medium: ≥5km and ≤10km; Far: >10km

** Low: <50 patients; Medium: ≥50 and ≤100 patients; High: >100 patients

### Effects of patient load of HIV care clinics on HIV transmission

Analysis of the overall population found that couples assigned to HIV care clinics with 50 to 100 patients or more than 100 patients per clinician had about 30% higher rate of transmission (unadjusted HR = 1·31, 95% CI, 1·21–3·52; HR = 1·29, 95% CI, 1·31–3·61, respectively) as compared to those at clinics where clinicians had lower patient loads (Table 5). After adjustment the effect in each level of patient load on HIV transmission rates increased (aHR = 1·46, 95% CI, 0·93–4·12; aHR = 1·50, 95% CI, 1·00–4·84, respectively), though measures became less precise.

**Table 5:** Assessment of the impact of healthcare access across tiers of care. Hazard ratios in Table A compare risk of transmission between couples assigned to clinics in which clinicians on average have between 50 and 100 (medium) or more than 100 patients (high), versus fewer than 50 patients. Hazard ratios in Table A compare risk of transmission among couples living between 5 and 10 km (medium) and more than 10 km (far), versus those living less than 5 km from their assigned clinic.

**A. Effect of the patient load per clinician at assigned clinic**				
	Exposure class*	Overall	Village tier	Town/County tier
Crude HR (95% CI)	Low	1·00	1·00	1·00
	Medium	1·31 (1·21–3·52)	1·45 (1·47–6·37)	1·56 (0·93–5·35)
	High	1·29 (1·31–3·61)	1·40 (1·54–5·72)	4·33 (0·04–13·89)
				
Adjusted HR^†^ (95% CI)	Low	1·00	1·00	1·00
	Medium	1·46 (0·93–4·12)	1·64 (0·82–5·73)	1·69 (0·78–6·12)
	High	1·50 (1·00–4·84)	1·55 (1·23–6·78)	4·45 (0·05–15·75)
**B. Effect of distance to assigned clinic**				
	Exposure class^‡^	Overall	Village tier	Town/County tier
Crude HR (95% CI)	Near	1·00	1·00	1·00
	Medium	1·35 (0·86–2·11)	1·60 (0·91–2·79)	0·20 (0·03–1·50)
	Far	0·46 (0·21–1·02)	1·11 (0·44–2·78)	0·10 (0·01–0·75)
				
Adjusted HR^†^ (95% CI)	Near	1·00	1·00	1·00
	Medium	1·22 (0·77–1·93)	1·83 (1·04–3·21)	0·19 (0·02–1·40)
	Far	0·50 (0·22–1·12)	1·13 (0·45–2·83)	0·10 (0·01–0·75)

HR: hazard ratio; CI: confidence interval

* Low: <50 patients; Medium: ≥50 and ≤100 patients; High: >100 patients

†Adjusted models included variables for clinic type, distance from clinic (if not already included as the exposure), patient volume (if not already included as the exposure), age, sex, and occupation. Stratified analyses show results of the unadjusted and adjusted models using interaction terms for clinic type (village level vs. town or county level).

‡ Near: <5km; Medium: ≥5km and ≤10km; Far: >10km

When stratified by clinic tier, unadjusted models of the impact of patient load per clinician on HIV transmission produced rate estimates that were 45% and 40% higher in mid-level and high patient loads, respectively, in those assigned to village clinics (HR = 1·45, 95% CI, 1·47–6·37; HR = 1·40, 95% CI, 1·54–5·72). A similar pattern followed in patients at township/county level clinics, though these measures of effect lacked precision. Following adjustment for confounding, transmission risk in each exposure category and across both tiers of care increased; however, only the measure among village level patients treated at the clinics with highest patient loads (100 or more patients per clinician) retained statistical significance (aHR = 1·55, 95% CI, 1·23–6·78; Table 5A).

### Effects of distance from HIV care center on HIV transmission

In analyses of the overall population, unadjusted Cox models showed that relative to living near (within 5 km) one’s assigned HIV clinic, those living between 5 and 10km had elevated rates of HIV transmission (HR = 1·35, 95% CI, 0·86–2·11) whereas those farthest away (over 10 km away) had lower rates (HR = 0·46, 95% CI, 0·21–1·02). Neither of these results was statistically significant. Following adjustment the effects of living a medium or farther distance from one’s assigned clinic only slightly moved each estimates closer towards the null (aHR = 1·22, 95% CI, 0·77–1·93; aHR = 0·50, 95% CI, 0·22–1·12, respectively).

Stratification by tier of care revealed differences in direction and magnitude of these effects. Whereas unadjusted models estimated that those living medium and farther distances from their village level clinics had elevated rates of HIV transmission (HR = 1·60, 95% CI, 0·91–2·79; HR = 1·11, 95% CI, 0·44–2·78); this same association was reversed among those assigned to township/county clinics (HR = 0·20, 95% CI, 0·03–1·50; HR = 0·10, 95% CI, 0·01–0·75). Only the protective effect of living beyond 10 km from one’s township/county clinics was statistically significant. Adjustment did not substantially alter stratified estimates, except in the case of those living medium distances from their village clinics among whom HIV transmission risk was 83% higher than those living close to their village clinics (HR = 1·83, 95% CI, 1·04–3·21; Table 5B).

## Discussion

We observed that patients assigned to HIV care clinics where clinicians faced high patient loads (>100 per person) had a higher risk of HIV transmission; this effect was enhanced at village clinics but less prominent at township and county clinics. Those treated at clinics with medium patient loads (10 to 50 patients per clinician) also had slightly elevated transmission risk, though this measure lacked precision. Regarding overall effects of travel distance, living at medium distances (between 5 and 10 km) from one’s assigned clinic increased HIV transmission risk, whereas living far (over 10 km) from one’s clinics was *protective against* transmission. Stratification identified even more extreme effects within tiers: the harmful effects of traveling medium distances were even more so when traveling to village clinics; similarly, protective effects of farther distances were even more beneficial when traveling to township or county level clinics.

The impact of high patient load on HIV transmission suggests that clinic resources and adequate visit time with clinicians play an important role in helping patients sustain viral suppression, especially in lower tier clinics. Adequate visit time is a vital to building stronger patient-provider relationships, a factor that has been linked to better health outcomes in HIV patients in the US.[7–9] Our finding that high patient load was even more detrimental against HIV prevention in village sites suggests that the burden of too many patients is experienced even more acutely by village doctors, who often work with fewer physical resources and training.

Distance decay, or the worsening of health outcomes with longer travel distance, is evident in the fact that those traveling medium distances faced higher transmission risk than those traveling the shortest distances. That this effect was amplified in patients traveling to village clinics suggests that geographical access to routine care may be as important as the quality of the care received once there. Reasons why those traveling *more than* 10 km for care at higher tier clinics had lower HIV transmission risk are unclear, but patients may value care from higher tier clinics differently, travel to which may be perceived as less of a burden. Although we did not formally assess respondents’ perceptions of care quality, other studies have found that Chinese HIV patients valued care from urban hospitals more than that from rural clinics,[27] and that distance was less of a burden to patients with concerns about the quality of care.[28,29] The link between patients’ confidence in their healthcare system and their tolerance for travel may therefore be a key mediator in predicting their treatment outcomes.

Two key features of this analysis inform interpretation of our results. First, distance was measured from patients’ homes to their *designated—*but not necessarily preferred—HIV care clinic. Distances for any patients seeking care at other clinics would therefore be mis-measured, though the direction of this bias or the extent of this problem is not assessable. Anecdotal evidence suggests that preferential clinic seeking outside of the referral chain is relatively uncommon in this study setting, likely due to the prohibitive cost burden of doing so. Second, we hypothesized that regular access to quality healthcare plays a reinforcing role in helping HIV patients sustain viral suppression through greater exposure to adherence counseling, earlier identification of virological failure, treatment of opportunistic infections, and maintenance of optimal drug regimens. We could not validate this hypothesis, however, because we lacked viral load data to link to our measures of healthcare access—patient volume and travel distance—with the outcome of HIV transmission risk. A final limitation in our assessment of association is potential for confounding bias residual from incomplete adjustment in our models.

Findings from this analysis support several modest recommendations in our study prefecture and other geographically similar regions. First, system wide improvements to reduce virological failure (and therefore HIV transmission events) would benefit from targeting of resources—additional support staff or specialized tools such as point-of-care viral load testing[30]—in particular for high volume, lower-tier clinics. Second, the outsized role of patients’ perceptions of care quality on their health outcomes could mean strategies to alter their perceptions, such as the aforementioned injection of resources into lower tier clinics, or through field visits or clinician trainings conducted by prominent HIV physicians, might not only improve care quality but also signal stronger intent by health officials to narrow disparities in quality.

As China’s rapidly developing healthcare system generates greater inequities in healthcare access,[31] emerging treatment as prevention programs may do well to prioritize attainable short term goals such as more responsive management of human resources and investment in specialized clinical technologies to streamline care. In ensuring that rural Chinese HIV patients attain the minimum standards intended by WHO treatment guidelines, we may do more to move the epidemic closer towards eradication by treatment.
